# Supplementary figures and images for: Differential activation of NLRP3 inflammasome by Acinetobacter baumannii strains
Source: PLoS One. 2022 Nov 1;17(11):e0277019. doi: 10.1371/journal.pone.0277019 (PMC9624416; doi:10.1371/journal.pone.0277019)

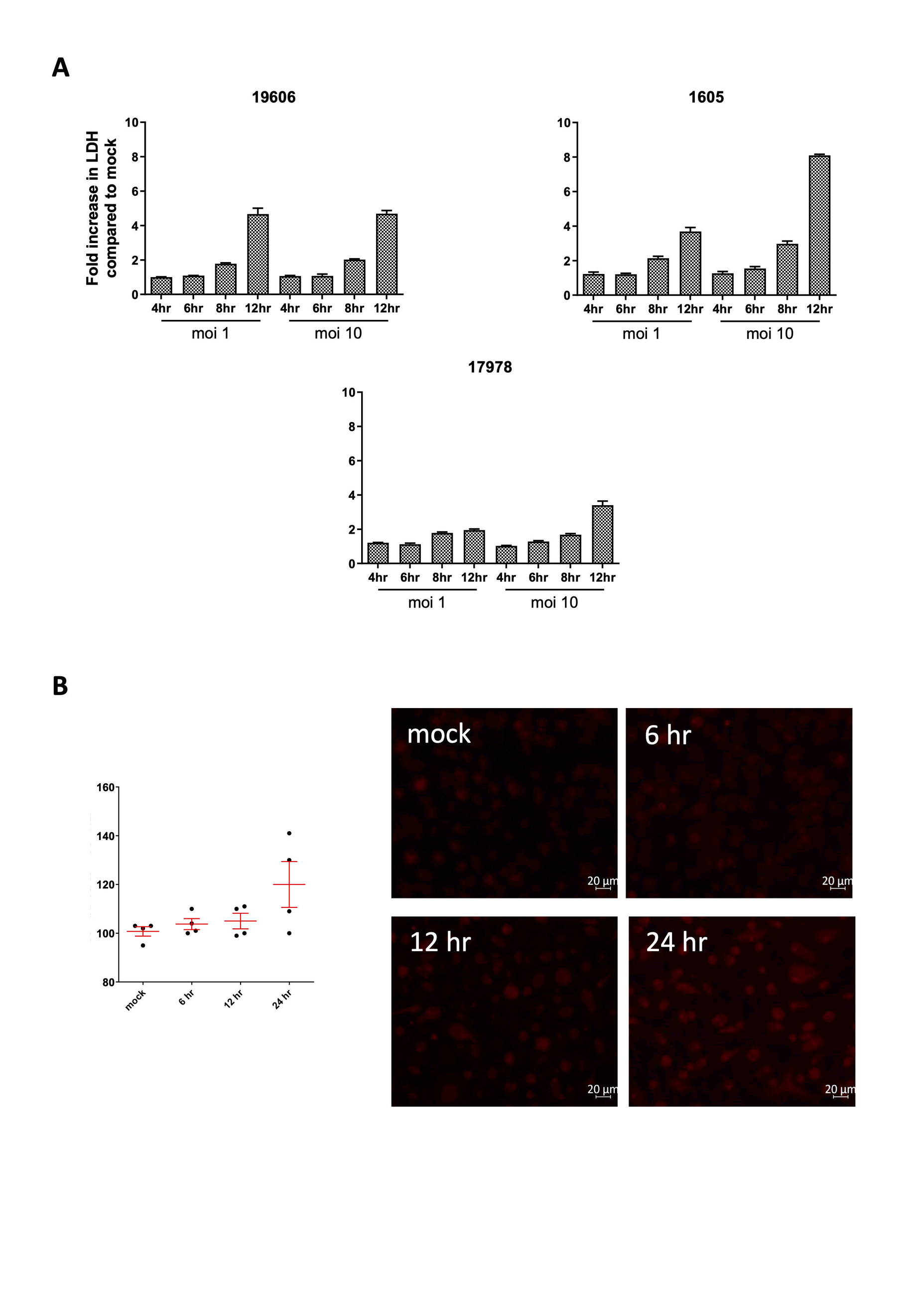

Supplement: S1 Fig — (A) LDH assay of mouse BMDM post A. baumannii infection. mean ± SEM. N = 3 per timepoint. (B) Zombie aqua quantification of mouse BMDM death post A. baumannii infection (strain ATCC BAA 1605). mean ± SEM. N = 4. (TIF) [file pone.0277019.s001.tif]

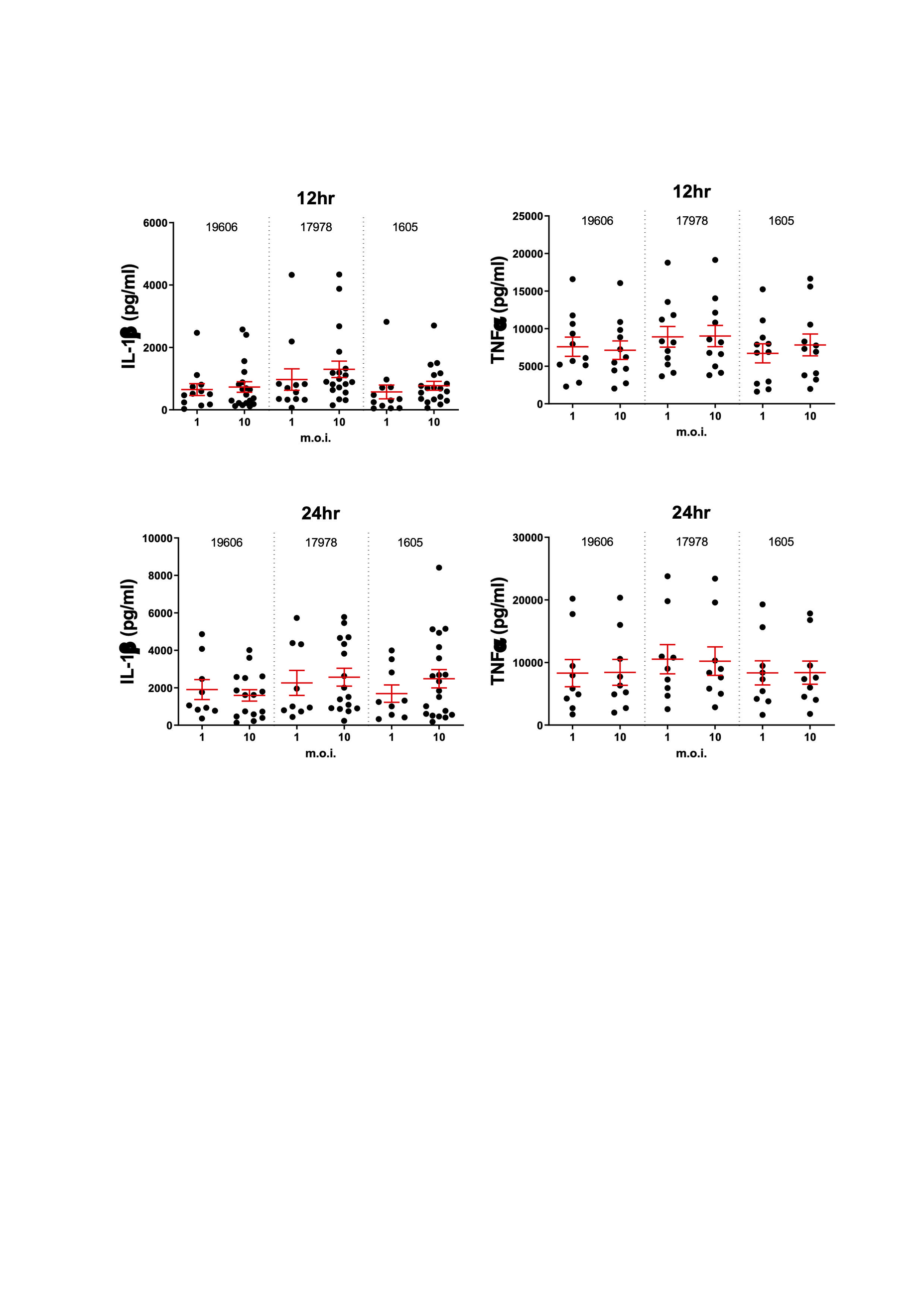

Supplement: S2 Fig — Mouse BMDMs were infected with indicated m.o.i. of live A. baumannii and analysed. ELISA of pro-inflammatory cytokines levels of TNFα and IL-1β produced by wild-type mouse BMDM after 12 and 24 hours of infection, n = 8 per strain. mean ± SEM. (TIF) [file pone.0277019.s002.tif]

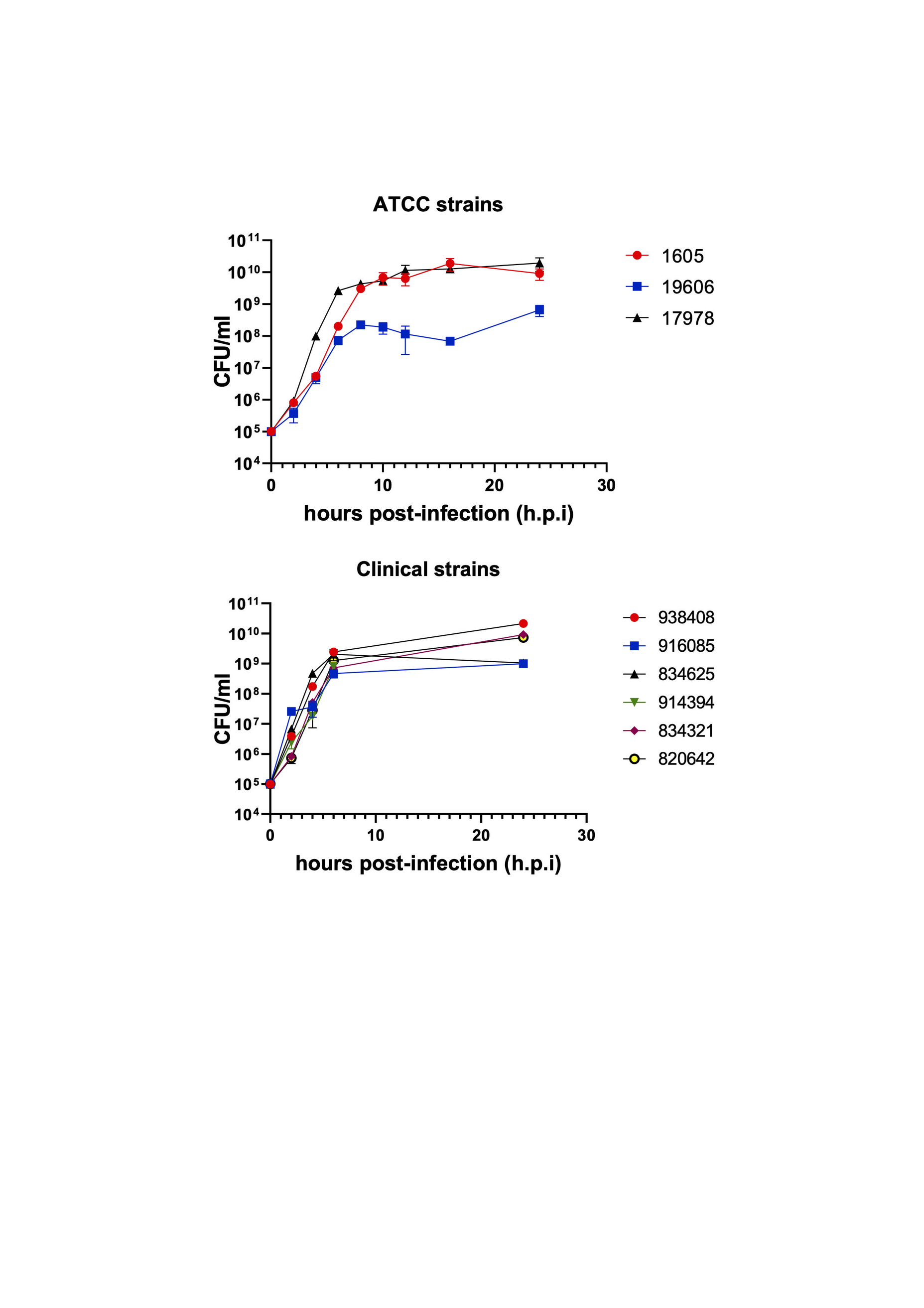

Supplement: S3 Fig — A. baumannii isolates growth rate (CFU/ml) up to 30 hours post inoculation. N = 7 per strain. All data are shown as mean ± SEM. (TIF) [file pone.0277019.s003.tif]

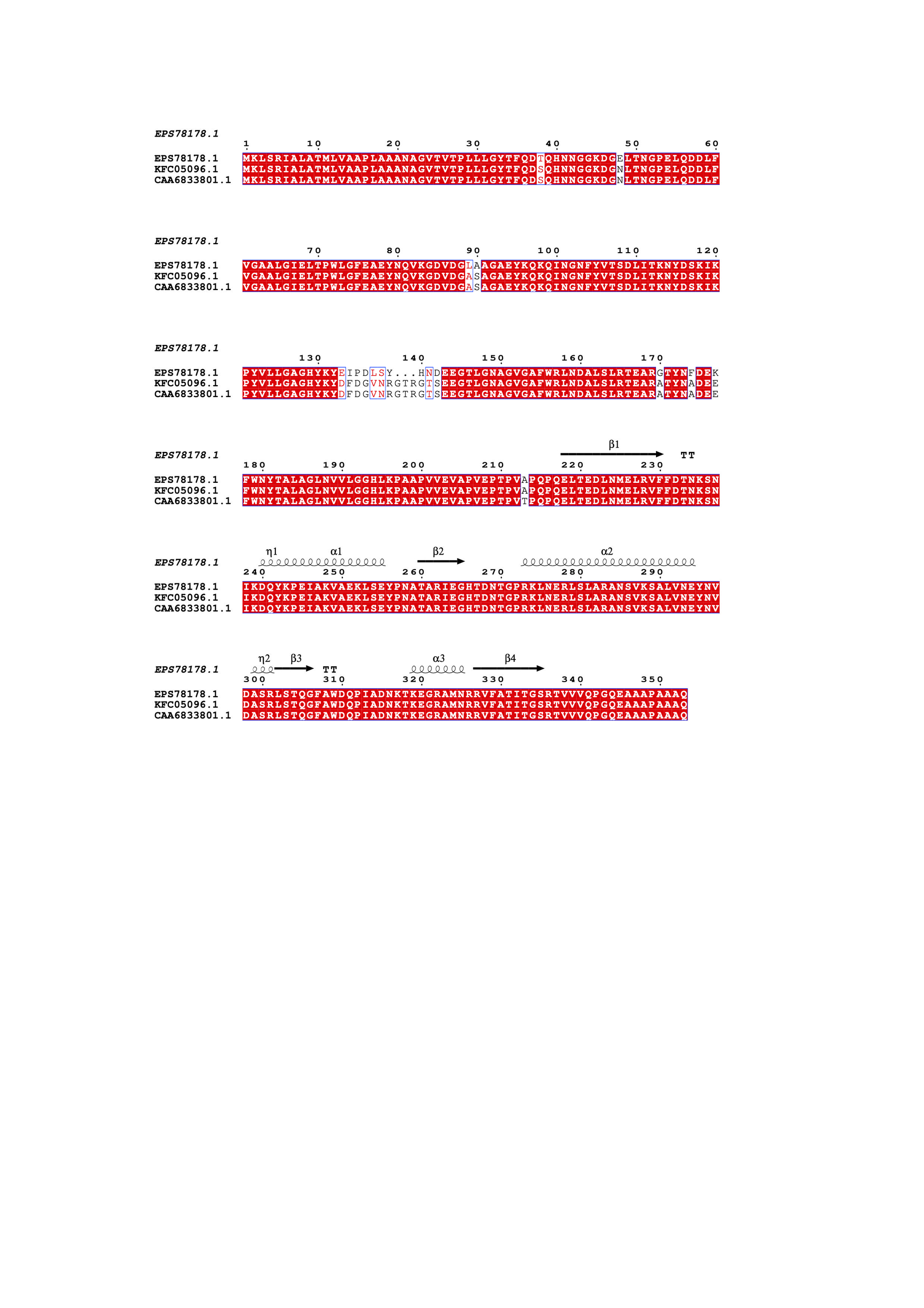

Supplement: S4 Fig — Protein sequences were aligned to Omp38 secondary structure and its OmpA-like domain (residues 221–339). The OmpA-like domain is fully conserved in all aligned protein sequences. (TIF) [file pone.0277019.s004.tif]
