# Supplementary material for: Differential activation of NLRP3 inflammasome by Acinetobacter baumannii strains
Source: PLoS One. 2022 Nov 1;17(11):e0277019. doi: 10.1371/journal.pone.0277019 (PMC9624416; doi:10.1371/journal.pone.0277019)
Supplement: S1 Table — (DOCX) [file pone.0277019.s005.docx]

**S1 Table. ATCC and clinical *A. baumannii* strain isolation sites and antibiotic resistance profiles**

| **Strain** | **Origin** | **Isolation site** | **Resistant to** |
| --- | --- | --- | --- |
| ATCC 19606 | ATCC | Urine | NA |
| ATCC 17978 | ATCC | Fatal meningitis | NA |
| ATCC  BAA-1605 (1605) | ATCC | Sputum | Ceftazidime, Gentamicin, Ticarcillin, Piperacillin, Aztreonam, Cefepime, Ciprofloxacin, Imipenem, Meropemem |
| 916085 | Canberra Hospital | Swab | Ciprofloxacin, Gentamicin, Meropenem, Tobramycin |
| 834625 | Canberra Hospital | Blood | Ceftriaxone, Trimethoprim |
| 938408 | Canberra Hospital | Blood | Amp/Amoxicillin, Amox/Clav Acid, Cefazolin |
| 820642 | Canberra Hospital | Blood | Amp/Amoxicillin, Amox/Clav Acid, Cefazolin, Ceftriaxone, Trimethoprim |
| 834321 | Canberra Hospital | Urine | Ceftriaxone, Ciprofloxacin, Gentamicin, Norfloxacin, Tobramycin, Meropenem |
| 914394 | Canberra Hospital | Urine | Amikacin, Cefepime, Ceftazidime, Ciprofloxacin, Gentamicin, Meropenem, Pip/Tazobactam, Tica/Clax Acid, Tobramycin |
