# Supplementary material for: Differential activation of NLRP3 inflammasome by Acinetobacter baumannii strains
Source: PLoS One. 2022 Nov 1;17(11):e0277019. doi: 10.1371/journal.pone.0277019 (PMC9624416; doi:10.1371/journal.pone.0277019)
Supplement: S2 Table — (DOCX) [file pone.0277019.s006.docx]

**S2 Table. Mouse primers used for qRT-PCR**

| **Name** | **Forward (5’-3’)** | **Reverse (5’-3’)** |
| --- | --- | --- |
| *Tnfa* | gcc tct tct cat tcc tgc ttg | ctg atg aga ggg agg cca tt |
| *Il1b* | gac ctt cca gga tga gga ca | agc tca tat ggg tcc gac ag |
| *Aim2* | gat tca aag tgc agg tgc gg | tct gag gct tag ctt gag gac |
| *Caspase-11* | aca atg ctg aac gca gtg ac | ctg gtt cct cca ttt cca ga |
| *Nlrp3* | gtg gtg acc ctc tgt gag gt | tct tcc tgg agc gct tct aa |
| *Nlrc4* | cta cat tga tgc tgc ctt gg | tct ctt cgt ctc tga gtc tc |
| *Gapdh* | gag gaa cct gcc aag tat g | tgg gag ttg ctg ttg aag |
