# Supplementary material for: Differential activation of NLRP3 inflammasome by Acinetobacter baumannii strains
Source: PLoS One. 2022 Nov 1;17(11):e0277019. doi: 10.1371/journal.pone.0277019 (PMC9624416; doi:10.1371/journal.pone.0277019)
Supplement: Attachment — Submitted filename: Response to reviewers_Plos one_revision2.docx [file pone.0277019.s008.docx]

Response to the reviewers

Reviewer #1: (No Response)

Reviewer #2: I raised points about analysing the available data (quantifying Western Blots) and characterizing the response at different timepoints, and the authors have adequately addressed by points and changed the manuscript accordingly.
I have two final comments:
1 – What is the purpose of the images in Supplementary Figure 1b? Are the images completely black because there is no signal? Or was there a problem in copying the images to the Word document?

**Response:** The mages in the supplementary data provide a cell death quantification by Zombie Aqua from 6 to 24 hours post inoculation. The images are representative field for quantification. We do apologise for the inconvenience. It seems the image conversion renders the images black. In the revised version, we have enhanced the contrast of the images to ensure these are not black.

2 – In Lines 323-324, authors need to correct the call to “Suppl Fig 1” to “Suppl Fig 2”.

**Response:** Many thanks for spotting this mistake. We have now fixed it.

Reviewer #3: (No Response)
